# Chimeric antigen receptor T-cell therapy for rheumatic diseases: from B-cell depletion to immune reset and beyond

**DOI:** 10.3389/fimmu.2026.1913968

**Published:** 2026-08-17

**Authors:** Yiqing Zhan, Chao Hu, Keyao Xiao

**Affiliations:** 1 Department of General Medicine, the Second Affiliated Hospital of Wannan Medical College, Wuhu, China; 2 Department of Gynecology, The First Affiliated Hospital of Wannan Medical College (Yijishan Hospital of Wannan Medical College), Wuhu, Anhui, China; 3 Department of Infectious Disease, Shaoyang Central Hospital, Shaoyang, China

**Keywords:** autoimmunity, B-cell depletion, CAR-Treg, chimeric antigen receptor T cells, idiopathic inflammatory myopathy, immune reset, systemic lupus erythematosus, systemic sclerosis

## Abstract

Rheumatic and autoimmune diseases have long relied on glucocorticoids and immunosuppressants for disease control, yet a true cure remains elusive; patients oscillate between remission and relapse and progressively accumulate irreversible organ damage. Chimeric antigen receptor (CAR) T cells targeting CD19 or B-cell maturation antigen (BCMA) achieve deep depletion of pathogenic B-lineage cells, enabling a subset of patients with systemic lupus erythematosus, idiopathic inflammatory myopathy, and systemic sclerosis to attain sustained remission after withdrawal of all immunosuppressants, accompanied by seroconversion of autoantibodies, normalization of complement, and reconstitution of the B-cell pool with a naive phenotype. This phenomenon suggests that, if B-cell depletion can reach the secondary lymphoid organs and inflamed tissues beyond the peripheral blood, it may “reset” the disrupted immune homeostasis rather than merely suppress it transiently. Compared with the oncology setting, the target-cell burden in autoimmune disease is lower, cytokine release syndrome is mostly mild and self-limiting, and neurotoxicity is rare; nevertheless, prolonged B-cell aplasia, hypogammaglobulinemia, and infection risk still require disciplined management, while signals of long-term secondary malignancy await validation through longer follow-up. Next-generation engineering strategies—including CD19/BCMA dual targeting, allogeneic universal products, CAR regulatory T cells (CAR-Treg), and logic gating with safety switches—are broadening indications, lowering costs, and enhancing precision and controllability. This review systematically examines the engineering principles, pharmacodynamics, cross-disease clinical evidence, distinctive safety profile, and frontier advances of CAR-T in rheumatic disease and discusses how this therapy may shift the treatment paradigm from “chronic suppression” toward a “one-time reset”, as well as the questions of durability, patient stratification, and health economics that must be answered on the path to a “cure.”

## 1 Introduction

Systemic autoimmune diseases are characterized by a sustained immune attack on the body’s own tissues, with clinical manifestations spanning multiple organ systems and a protracted disease course. Taking systemic lupus erythematosus (SLE) as an example, its diagnosis and classification have long depended on an integrated clinical and serological judgment; the 2019 European League Against Rheumatism/American College of Rheumatology (EULAR/ACR) classification criteria set antinuclear antibody positivity as an entry requirement, underscoring the central role of adaptive immune activation and autoantibody formation in defining the disease (1, 2). However, the updating of classification criteria has not altered one fundamental dilemma: current therapies can mostly only control, but not eradicate, the disease.

### 1.1 The therapeutic dilemma in rheumatic diseases

The current standard of care for rheumatic disease is built on glucocorticoids, antimalarials, conventional synthetic disease-modifying antirheumatic drugs, and biologics, whose shared logic is to suppress the intensity of the immune response rather than to correct its direction. Long-term, broad-spectrum immunosuppression brings cumulative toxicities such as infection, osteoporosis, metabolic disturbance, and cardiovascular events, while the damage inflicted by the disease on the vasculature, kidneys, lungs, and skin is often irreversible (3–6). For refractory patients, repeated relapses mean ongoing loss of organ function and high medical and societal costs. Even targeted agents with established efficacy, such as belimumab, which inhibits B-lymphocyte stimulator (BLyS), also known as B-cell–activating factor (BAFF), reduced disease activity and relapses in the two pivotal phase III trials BLISS-52 and BLISS-76, yet its effect is suppressive rather than curative and requires long-term maintenance dosing (7–11). In lupus nephritis, the BLISS-LN trial further confirmed that belimumab added to standard therapy improves renal response rates, but the attainment and maintenance of remission still depend on uninterrupted drug exposure (12). This “control dependence” is precisely the impetus for a paradigm shift.

### 1.2 The central role of the B-cell lineage in autoimmunity

Placing B cells back at the center of autoimmune pathogenesis is one of the most important conceptual shifts in immunology over the past two decades. B cells are far more than antibody factories: as highly efficient antigen-presenting cells they participate in initiating follicular helper T cell (Tfh) and germinal center responses (13–18); they secrete a range of pro-inflammatory and regulatory cytokines, including IL-6, TNF, and GM-CSF, directly shaping the local inflammatory microenvironment (19–21); and within inflamed tissues they drive the formation of ectopic lymphoid structures (tertiary lymphoid structures), sustaining *in situ* presentation of autoantigens and expansion of autoreactive clones at the lesion site (22, 23). In SLE, aberrant activation of autoreactive B cells drives autoantibody production and epitope spreading, and specific subsets such as double-negative 2 (DN2) B cells are closely associated with disease activity (24–29). Notably, the benefit of B-cell depletion often exceeds its effect on autoantibody titers—clearing pathogenic B cells can downregulate type I interferon signaling in monocytes and T cells, indicating that B cells occupy an upstream hub position in the autoimmune inflammatory network (30–34).

### 1.3 From the limitations of anti-CD20 monoclonal antibodies to the leap of CAR-T

B-cell-depleting therapy is not a new concept. Rituximab, a type I anti-CD20 chimeric monoclonal antibody, depletes circulating B cells through complement-dependent and antibody-dependent cytotoxicity, yet it failed to meet the primary endpoint in both of its randomized controlled trials in SLE—EXPLORER (non-renal) and LUNAR (lupus nephritis) (35, 36). The failure has been attributed in part to trial design, but a deeper biological explanation is that anti-CD20-mediated depletion is “superficial”—it struggles to fully eliminate tissue-resident B cells, is powerless against CD20-negative plasmablasts and long-lived plasma cells, and achieves limited depth and durability of peripheral depletion (37–39). The glycoengineered type II anti-CD20 antibody obinutuzumab achieves deeper and more durable B-cell depletion through enhanced antibody-dependent cytotoxicity, significantly improving renal response rates in lupus nephritis in the NOBILITY and REGENCY trials, indirectly corroborating the hypothesis that “depth of depletion determines efficacy” (40–42).

The fundamental breakthrough of CAR-T lies in the “depth” and “breadth” of its depletion. CD19 is expressed not only on mature B cells but also on plasmablasts and some early plasma cells, and as living, proliferative effector cells, CAR-T can actively infiltrate secondary lymphoid organs and inflamed tissues (43, 44). Multiple studies have shown that CD19 CAR-T can achieve near-complete depletion of B-lineage cells in both peripheral blood and tissues, far exceeding what anti-CD20 antibodies can attain (45). This deep depletion lays the biological foundation for “immune reset”—that is, reconstitution of the immune repertoire by naive, non-autoreactive B cells after pathogenic clones have been eradicated (46, 47).

### 1.4 The differing contest in cancer versus autoimmunity

Translating CAR-T from cancer to autoimmunity requires a renewed understanding of the biological context of its action. In B-cell malignancies, the target-cell burden can reach hundreds of grams to kilograms, and the vigorous *in vivo* expansion of CAR-T often triggers severe cytokine release syndrome and neurotoxicity (48–50). In autoimmune disease, by contrast, the total burden of pathogenic B cells is far lower, and the cytokine elevation provoked by CAR-T expansion is correspondingly mild, manifesting clinically as mostly grade 1, self-limiting cytokine release syndrome (51). Moreover, the inflammatory context of autoimmunity differs markedly from the tumor microenvironment and lacks the immunosuppressive milieu characteristic of tumors, which influences both the expansion kinetics of CAR-T and the requirement for its persistence—in the autoimmune setting, brief but sufficient depletion may already suffice to achieve reset, without the long-term persistence pursued in cancer therapy (52). Together, these differences make autoimmune disease a relatively “ideal” setting for CAR-T application, with a wider safety window.

## 2 Engineering principles and pharmacodynamics of CAR-T in Rheumatic diseases

### 2.1 The design philosophy of CAR architecture

**Figure 1:**
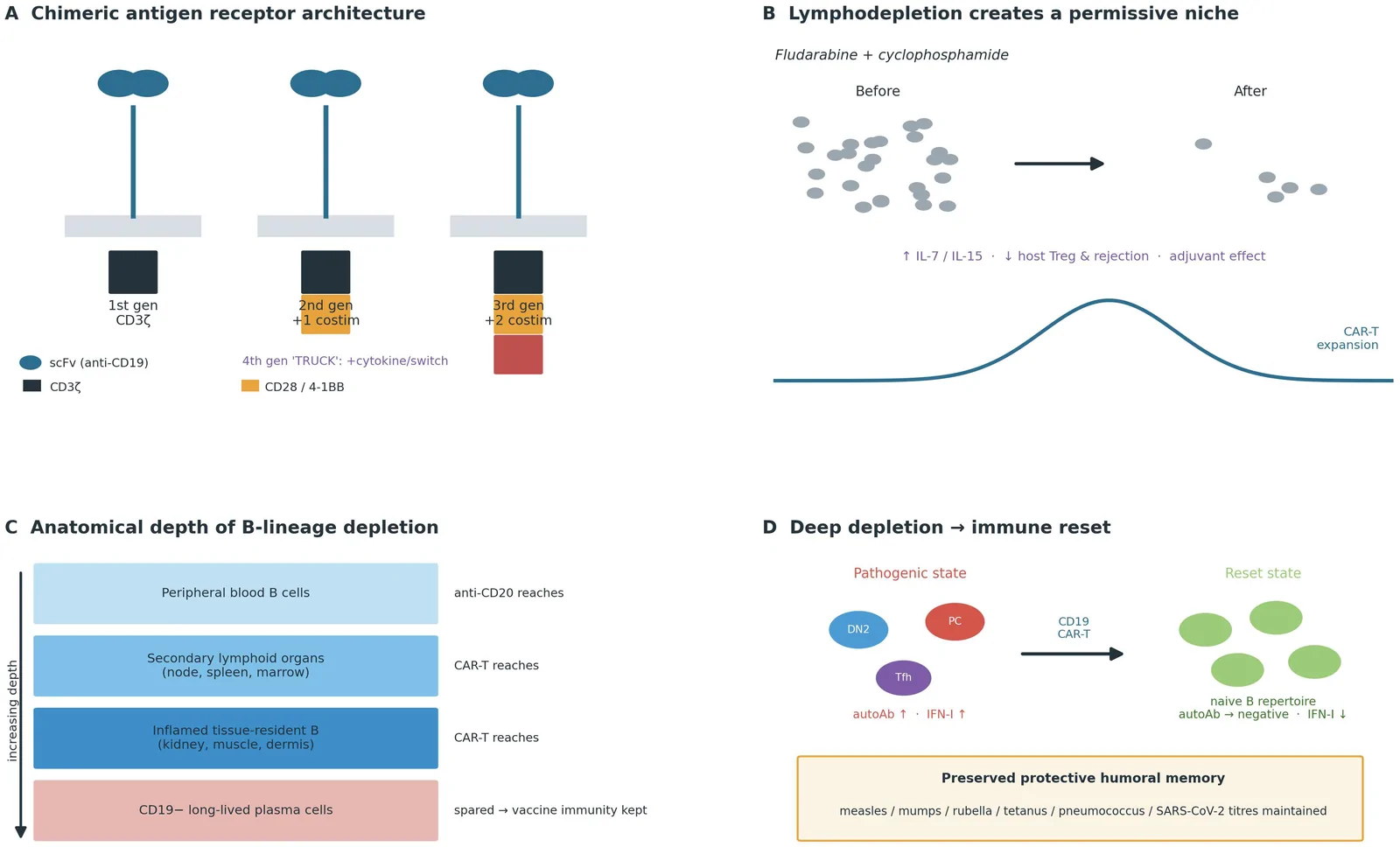
Engineering principles and the mechanistic panorama of CAR T-cell therapy in autoimmunity. **(A)** Chimeric antigen receptor architecture across generations. **(B)** Lymphodepletion creates a permissive niche. **(C)** Anatomical depth of B-lineage depletion. **(D)** Deep depletion drives immune reset while protective humoral memory is preserved.

The chimeric antigen receptor is a class of synthetic fusion receptors whose extracellular portion is typically a single-chain variable fragment (scFv) that recognizes the target antigen, connected through a hinge region and a transmembrane domain to the intracellular signaling region (53–56). First-generation CARs contained only the CD3ζ activation domain and showed insufficient expansion and persistence; second-generation CARs introduced one costimulatory domain (4-1BB or CD28), markedly improving *in vivo* survival and effector function and constituting the bulk of current clinical products; third-generation CARs link two costimulatory domains in tandem (57–63). The choice of costimulatory domain finely shapes CAR-T behavior: CD28-mediated signaling is more rapid and produces stronger effector function but is more prone to exhaustion, favoring an effector-memory phenotype, whereas 4-1BB signaling is slower and more conducive to the formation of central-memory phenotypes and long-term survival (64–67). In the autoimmune setting, one study observed that a product using the CD28 costimulatory domain (KYV-101) induced CAR-T cells with an effector-memory/terminally differentiated effector-memory re-expressing CD45RA (TEMRA) phenotype accompanied by upregulated CXCR3 expression, suggesting that these cells possess Th1-polarized features and strong tissue-infiltrating capacity—the latter possibly being especially critical for clearing tissue-resident B cells (68). The affinity of the scFv and the flexibility of the hinge region likewise influence synapse formation between the CAR and target cells, signaling strength, and even off-target risk, making them key engineering parameters for balancing efficacy and safety (69–72) (**Figure 1**).

### 2.2 Rethinking lymphodepleting preconditioning

Lymphodepleting (conditioning) preconditioning prior to CAR-T infusion is a necessary step for therapeutic efficacy. Standard regimens mostly use fludarabine combined with cyclophosphamide, with three mechanisms of action: depleting endogenous lymphocytes to free up “immune space” and cytokines (such as IL-7 and IL-15) for CAR-T expansion; weakening the host immunity that might reject the CAR-T; and enhancing CAR-T activation through an adjuvant-like effect (73–75). However, rheumatic patients have been chronically exposed to immunosuppressants, and their marrow reserve and immune status differ from those of treatment-naive cancer patients, so excessively intense lymphodepletion may bring unnecessary myelosuppression and infection risk. Therefore, in the autoimmune setting, most regimens adopt a reduced-intensity fludarabine/cyclophosphamide combination to control toxicity while ensuring adequate CAR-T expansion (76). How to individualize the optimization of lymphodepletion intensity for rheumatic patients with differing backgrounds of organ damage remains a question that urgently requires prospective data.

### 2.3 The anatomical hierarchy of deep B-cell depletion

The core of CAR-T efficacy lies in the well-defined anatomical hierarchy of its depletion “depth.” The shallowest level is the depletion of peripheral blood B cells, which anti-CD20 antibodies can also achieve; the advantage of CAR-T is manifested at deeper levels—B cells within the secondary lymphoid organs (lymph nodes, spleen, bone marrow) and B-lineage cells residing in inflamed tissues (the glomeruli in lupus nephritis, the endomysium in myositis, the dermis in sclerosis) (77, 78). As living cells capable of active migration and proliferation, CAR-T can track down and clear the pathogenic cells in these “sanctuaries” that antibody therapies struggle to reach (79, 80). Clinical observations show that after CD19 CAR-T therapy, peripheral blood B cells fall to undetectable levels within days, and this depletion is equally thorough at the tissue level, providing a “blank-slate” state for subsequent immune reconstitution (81).

### 2.4 Immune reset: the ultimate manifestation of the therapeutic effect

“Immune reset” is the most striking concept of CAR-T in the field of autoimmunity and is also what fundamentally distinguishes it from all previous B-cell-targeted therapies. After deep depletion, B cells typically begin to reconstitute within months, but the reconstituted B-cell pool exhibits a fundamental change: the overwhelming majority are naive B cells, class-switched memory B cells and plasmablasts are durably absent, and the B-cell receptor repertoire displays a “primary, “ antigen-inexperienced signature (82). In SLE patients, the disease-associated DN2 cell population remains persistently reduced, and autoreactive clones disappear from the reconstituted repertoire (24). Importantly, the protective antibodies maintained by long-lived plasma cells (such as those against measles, mumps, rubella, tetanus, pneumococcus, and SARS-CoV-2 vaccines) remain relatively stable after treatment, indicating that CD19-negative long-lived plasma cells in the bone marrow can escape CD19 CAR-T depletion, thereby preserving pre-existing immune memory while resetting pathogenic humoral immunity (83, 84).

The impact of immune reset is not confined to B cells themselves. With the depletion of pathogenic B cells, the downstream immune network is reprogrammed: Tfh-driven germinal center responses weaken, B-cell clones expressing IgG and IgA nearly disappear while IgM/IgD clones expand, and type I interferon signaling in monocytes and T cells is markedly downregulated (30). This series of changes mechanistically supports a causal relationship between the B-cell response and the interferon signature in SLE, and also explains why the clinical benefit of B-cell depletion can exceed a simple decline in autoantibodies (31). The rebalancing of the Tfh, Th17, and Treg networks is thought to be the immunological basis for the maintenance of drug-free remission (85, 86). Viewed more broadly, B cells occupy a dual position in autoimmunity, functioning simultaneously as core drivers of pathology and as accessible network nodes for systemic intervention; this framing lends conceptual support to the notion that their deep depletion can reset, rather than merely dampen, a dysregulated immune system (87).

### 2.5 A mechanistic showdown with conventional B-cell-directed therapies

Placing CAR-T within the spectrum of B-cell-targeted therapies allows its uniqueness to be appreciated more clearly. Rituximab and obinutuzumab deplete CD20-expressing B cells through antibody effector mechanisms, but they are ineffective against plasmablasts and plasma cells, achieve limited tissue depletion, and do not erase immune memory (88). Belimumab inhibits B-cell survival and maturation by neutralizing BAFF, acting more indirectly and primarily affecting peripheral transitional and naive B cells, with minimal effect on established autoreactive memory and plasma cells (11). The proteasome inhibitor bortezomib can deplete plasma cells and thereby lower autoantibodies, but it lacks the capacity to remodel the B-cell repertoire and has a different toxicity profile (89, 90). By contrast, CAR-T achieves, in the form of living cells, deep, cross-tissue depletion of the entire B lineage (including plasmablasts) and, through the subsequent naive-repertoire reconstitution, erases immune memory—herein lies the mechanistic uniqueness that enables it to induce drug-free remission. This review uses **Table 1** to systematically compare the above therapies across dimensions such as target, depth of depletion, tissue penetrance, and capacity to erase immune memory.

**Table 1:** Mechanistic comparison of CAR T-cell therapy with conventional B-cell-directed agents.

Agent	Target	Mechanism	Depth (blood/tissue)	Plasma cells	Repertoire reset	Dosing
CD19 CAR-T	CD19	Living cytotoxic effector	Deep: blood + tissue + lymphoid organs	Plasmablasts + CD19+ early PC	Yes (naive reset)	Single
Rituximab	CD20	ADCC/CDC antibody	Peripheral; limited tissue	Spared	No	Repeated
Obinutuzumab	CD20 (type II)	Enhanced ADCC	Deeper than rituximab; still antibody	Spared	No	Repeated
Belimumab	BAFF/BLyS	Neutralizes survival factor	Indirect; transitional/naive	Minimal effect	No	Chronic
Bortezomib	Proteasome	Proteasome inhibition	Systemic	Depletes plasma cells	No	Cycles
BCMA CAR-T	BCMA	Living cytotoxic effector	Deep: bone-marrow plasma-cell niche	Long-lived plasma cells depleted	No† (CD19^+^ B cells largely spared)	Single
CD19/BCMA dual CAR-T	CD19 + BCMA	Living cytotoxic effector	Deep: B lineage + plasma cells	Plasmablasts + long-lived plasma cells	Yes (naïve reset)	Single
BCMA T-cell engager	BCMA (×CD3)	Bispecific antibody (off-the-shelf)	Peripheral; transient	Plasma cells reduced while dosed	No	Repeated

The “Repertoire reset” column indicates whether a therapy induces durable qualitative remodelling of the B-cell repertoire—reconstitution of a naïve-predominant B-cell pool accompanied by the disappearance of autoreactive and class-switched memory clones and a primary, antigen-inexperienced B-cell-receptor signature (as defined in Section 2.4)—rather than transient numerical depletion. “Yes” is assigned only where this qualitative reset has been demonstrated; “No” denotes depletion without durable repertoire remodelling. The determination therefore rests not on immature B-cell reconstitution alone, but on the combined disappearance of autoreactive/memory clones and the naïve character of the reconstituted repertoire. †For BCMA-directed CAR-T, plasma-cell depletion is achieved but CD19^+^ naïve and memory B cells are largely spared, so a full naïve repertoire reset is not established. ADCC, antibody-dependent cellular cytotoxicity; BAFF, B-cell–activating factor; BLyS, B-lymphocyte stimulator; CDC, complement-dependent cytotoxicity; PC, plasma cell.

## 3 Clinical translation: a spectrum of evidence from pioneering cases to disease modification

Within just a few years, the clinical evidence for CAR-T in autoimmune disease has rapidly accumulated from single case reports into case series and early clinical trials spanning multiple disease entities. In 2021, Mougiakakos et al. first reported marked improvement in a patient with refractory SLE after CD19 CAR-T therapy, opening up this field (91). At the time of writing, the number of autoimmune patients treated with CAR-T worldwide remains limited—approximately several dozen peer-reviewed published cases, with the global total estimated to be still on the order of a few hundred—yet the strength of its efficacy signal is already sufficient to reshape the field’s expectations (92). It should be emphasized that the existing evidence is predominantly from small-sample, single-arm, open-label studies and lacks randomized controlled data; the robustness of the conclusions awaits testing in larger cohorts and longer follow-up.

### 3.1 Systemic lupus erythematosus — a paradigm proof of drug-free remission

#### 3.1.1 Milestone cases and expanded cohorts

SLE is the disease entity with the most substantial evidence for CAR-T in autoimmunity. In 2022, Mackensen et al. reported the results of five refractory SLE patients treated with autologous CD19 CAR-T under a compassionate-use framework: the CAR-T expanded adequately *in vivo*, induced deep B-cell depletion, improved clinical symptoms, normalized laboratory indices, and caused only mild cytokine release syndrome; the reconstituted B cells displayed a naive, non-class-switched phenotype (51, 93). This pioneering study was subsequently expanded. In 2024, Müller et al. reported in the New England Journal of Medicine the follow-up results of 15 patients with systemic autoimmune disease (8 with SLE, 3 with idiopathic inflammatory myopathy, and 4 with systemic sclerosis) treated with CD19 CAR-T, confirming deep, cross-disease remission with manageable safety (82). The body of work from the Erlangen team in Germany laid the core evidentiary foundation for CAR-T in rheumatic disease.

#### 3.1.2 Depth of clinical and serological remission

The response of SLE patients to CD19 CAR-T is renowned for its “depth.” Many patients achieved drug-free remission after treatment, meeting the DORIS (Definition Of Remission In SLE) criteria, with SLEDAI scores falling to zero, sustained seroconversion of anti-double-stranded DNA antibodies, and normalization of complement C3/C4 (94). Crucially, this remission was attained and maintained after withdrawal of all glucocorticoids and immunosuppressants, and follow-up has shown it can be sustained for years—far exceeding the “drug-free” duration achievable by any previous therapy (95). This phenomenon directly challenges the traditional notion that “SLE requires lifelong treatment.”

#### 3.1.3 Histological remission

Remission is reflected not only at the serological level. In lupus nephritis patients, repeat renal biopsy after treatment showed regression of glomerular proliferative lesions and reduced or even absent immune-complex deposition, indicating that the depleting action of CAR-T indeed reached the kidney—a key target organ—and reversed the local immunopathology (96). Improvement at the histological level provides the most compelling evidence that “CAR-T can achieve disease modification rather than merely symptom relief.”

#### 3.1.4 The Chinese experience with the BCMA-CD19 dual-target strategy

Chinese investigators have made important contributions to the dual-target strategy. Early exploration proposed using a BCMA-CD19 compound CAR to simultaneously target mature B cells and plasma cells (97–99). In 2024, Wang et al. reported in the Annals of the Rheumatic Diseases a phase I open-label trial of BCMA-CD19 compound CAR-T for SLE, showing that the strategy was safe with considerable efficacy and that most patients achieved remission within 3 months (100). A phase I trial of co-infused CD19 and BCMA CAR-T further confirmed that the combination was safe in adults with refractory lupus, with most patients achieving remission after 3 months (101, 102). The theoretical advantage of dual targeting is that CD19 covers mature B cells and plasmablasts while BCMA additionally clears long-lived plasma cells, thereby eliminating the sources of autoantibodies that single-target CD19 could not reach and achieving a more thorough reset of humoral immunity (103, 104). The cost is deeper humoral immunosuppression, requiring a trade-off between infection risk and the preservation of protective vaccine antibodies.

### 3.2 Idiopathic inflammatory myopathy — biological restoration of muscle strength

#### 3.2.1 From wheelchair to walking

Idiopathic inflammatory myopathy (IIM) is one of the disease entities with the most dramatic responses to CAR-T. In refractory cases such as antisynthetase syndrome, CD19 CAR-T therapy brought about substantial recovery of muscle strength—reversal of manual muscle test scores, a cascading decline in creatine kinase levels, and the regaining of the ability to walk in some patients who had previously been wheelchair-dependent (105–109). Pecher et al. reported in JAMA a case of CD19 CAR-T treatment for antisynthetase syndrome-associated myositis and interstitial lung disease, documenting concurrent improvement in muscle strength and pulmonary function (110). The refractory antisynthetase syndrome case reported by Müller et al. in The Lancet, together with the report by Taubmann et al. on CD19 CAR-T salvage therapy after the failure of multiple B-cell-depleting antibodies, further enriched the evidence for IIM (68, 111).

#### 3.2.2 Disappearance of autoantibodies and switching-off of muscle MHC class I expression

Mechanistically, after CAR-T therapy myositis-specific autoantibodies disappeared, accompanied by a decline in the aberrantly upregulated MHC class I expression on muscle fibers—the latter being a hallmark of autoimmune attack during active myositis, and its “switching-off” being tantamount to “cutting the power” to the ongoing immune assault (112, 113). This reversal at the level of muscle pathology coincided temporally with the clinical recovery of muscle strength, supporting the view that CAR-T acts on the upstream driving mechanism of the disease.

#### 3.2.3 Concurrent improvement of interstitial lung disease

Antisynthetase syndrome is often complicated by progressive interstitial lung disease, a major determinant of poor prognosis. Of note, while improving the muscle disease, CD19 CAR-T therapy was accompanied in some patients by concurrent improvement of interstitial lung disease on imaging and pulmonary function, suggesting that the therapy also acts on the fibrotic inflammatory process involving the lungs (114).

### 3.3 Systemic sclerosis — a dawn for the reversal of fibrosis

#### 3.3.1 Rapid softening of skin sclerosis

Systemic sclerosis (SSc) is characterized by progressive fibrosis of the skin and internal organs and has long lacked effective means to reverse fibrosis. In 2023, Bergmann et al. first reported the result of a patient with severe SSc treated with CD19 CAR-T (115–117). Subsequently, Auth et al. reported in The Lancet Rheumatology a case series of diffuse SSc, showing a marked decline in the modified Rodnan skin score (mRSS), relatively rapid softening of skin sclerosis after treatment, and improvement in patient-reported overall health (118). For refractory, life-threatening SSc patients who are unsuitable for autologous hematopoietic stem cell transplantation, CAR-T offers a new option to modulate immunity and potentially reverse disease progression (119). Merkt et al. additionally reported an exploration of third-generation CD19.CAR-T-based combination therapy for Scl70-positive SSc (120).

#### 3.3.2 Radiological reversal of visceral fibrosis

More significant is the improvement of visceral involvement. Some cases showed improvement in myocardial T1-mapping parameters and regression of ground-glass opacities on pulmonary high-resolution CT after treatment, suggesting that the benefit of CAR-T extends to key internal organs such as the heart and lungs (121). Given the high lethality of visceral fibrosis in SSc, such radiological reversal signals, if reproducible in larger cohorts, would be of major clinical value. It should be noted that hematopoietic stem cell transplantation studies in SSc (such as the ASTIS and SCOT trials) have demonstrated that potent immune reset can alter the course of SSc, providing a conceptual precedent for the application of CAR-T in this disease (122, 123).

#### 3.3.3 B-cell–fibroblast crosstalk

In SSc, B cells drive the fibrotic process by secreting pro-fibrotic cytokines such as IL-6 and TGF-β and by interacting directly with fibroblasts (124–126). After CD19 CAR-T depletes B cells, this pro-fibrotic cytokine environment is interrupted, indirectly suppressing fibroblast activation and providing a mechanistic explanation for the reversal of fibrosis (127). The randomized controlled evidence for rituximab in improving SSc skin involvement, together with the results of the phase III focuSSced trial of tocilizumab (anti-IL-6 receptor), lend collateral support to the role of the B-cell–IL-6 axis in SSc fibrosis (128, 129).

### 3.4 Expanding the frontier

The boundaries of CAR-T application are expanding to more rheumatic and autoimmune disease entities. Early exploration has occurred in ANCA-associated vasculitis, Sjögren’s syndrome, and rheumatoid arthritis (130, 131). Haghikia et al. reported a patient with rheumatoid arthritis complicated by myasthenia gravis who, after CD19 CAR-T, showed clinical improvement and autoantibody seroconversion (132). In the field of neuroimmunology, CD19 CAR-T has been used in autoantibody-driven diseases such as multiple sclerosis and myasthenia gravis, and a BCMA/CD19 dual-target strategy achieved drug-free remission in most patients with refractory myasthenia gravis (133–138). These cross-disciplinary explorations share a common logic: any disease driven by autoantibodies or pathogenic B cells and poorly responsive to conventional depletion therapy could theoretically benefit from deep B-cell depletion and immune reset. The selection of indications should therefore be based on the B-cell dependence and refractoriness of the disease, rather than on traditional organ-specialty boundaries.

### 3.5 Cross-disease comparison of response patterns

**Figure 2:**
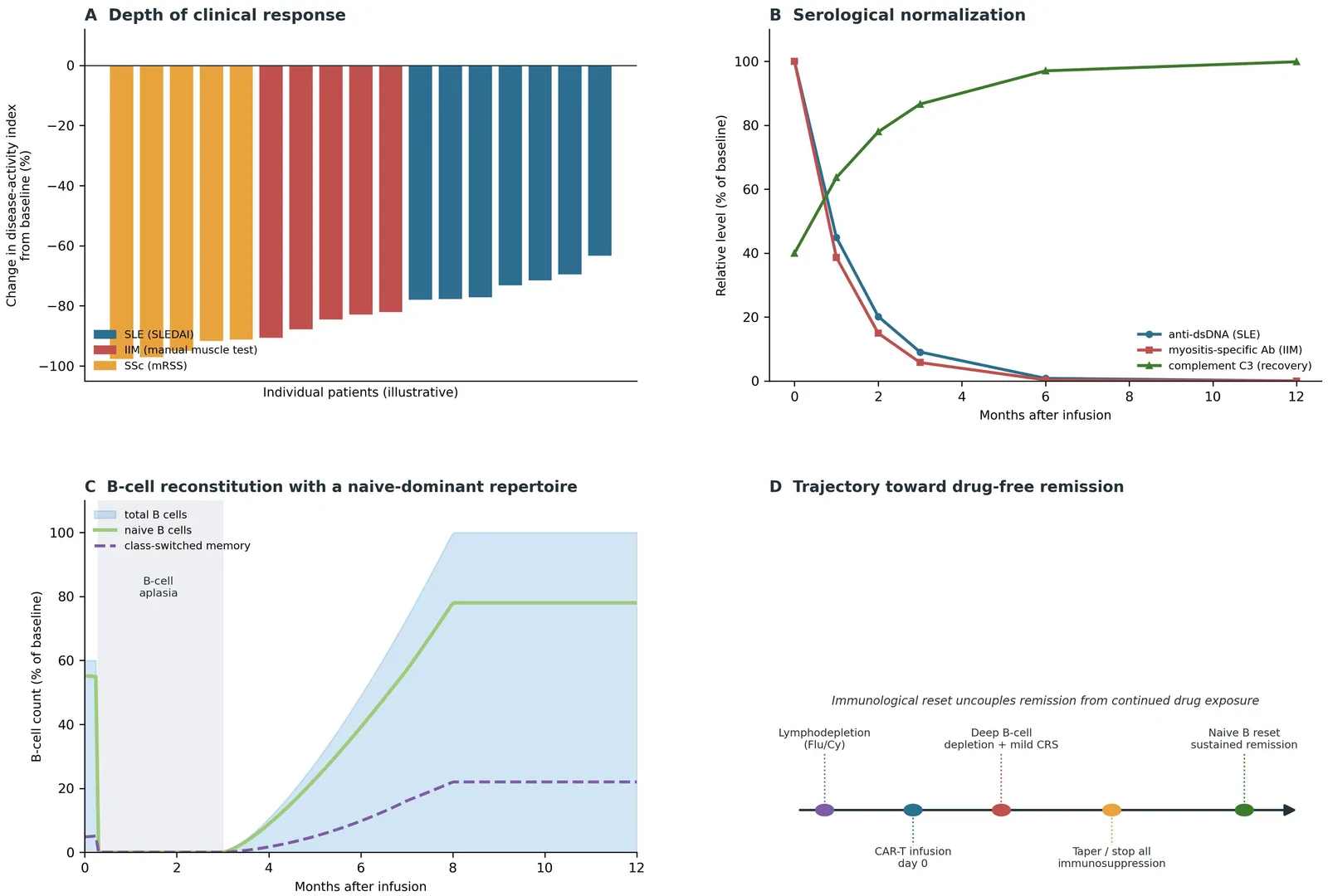
Clinical response patterns across SLE, IIM and SSc (illustrative schematic; not patient-level data). **(A)** Depth of clinical response. **(B)** Serological normalization. **(C)** Naive-dominant B-cell reconstitution. **(D)** Trajectory toward drug-free remission.

A side-by-side comparison of the responses in SLE, IIM, and SSc reveals that the core immunological changes are highly consistent: clearance of autoantibodies, reconstitution of the B-cell pool with a naive phenotype, and remodeling of T-cell subsets (82). This consistency suggests that, regardless of clinical phenotype, the immunological “common pathway” of CAR-T action is the same. At the same time, tissue-level responses show disease-specific differences: SLE is characterized by rapid serological and renal histological remission, IIM is marked by recovery of muscle strength and muscle enzymes, and SSc manifests as gradual reversal of fibrosis—the last being relatively slow because it involves already-established structural changes (45). This pattern of “immunological consistency, histological divergence” has direct implications for the design of assessment time windows and the choice of endpoints in evaluating efficacy. This review uses **Table 2** to summarize the major published and registered clinical evidence, and **Table 3** to compare the response characteristics across diseases from multiple dimensions (**Figure 2**). 

**Table 2:** Representative published clinical evidence of CAR T-cell therapy in rheumatic and autoimmune disease.

Study (year)	Design / n	Indication	CAR target	Key reported outcome	Safety
Mougiakakos (2021)	Case report, n=1	Refractory SLE	CD19	First proof-of-concept remission	Mild
Mackensen (2022)	Compassionate use, n=5	Refractory SLE	CD19	Drug-free remission; DORIS; naive B-cell return	Grade 1 CRS
Mueller (2024, NEJM)	Case series, n=15	SLE/IIM/SSc	CD19	Sustained drug-free remission across diseases	Mostly grade 1 CRS; no severe ICANS
Pecher (2023, JAMA)	Case	Antisynthetase IIM + ILD	CD19	Muscle and pulmonary improvement	Manageable
Bergmann (2023)	Case, n=1	Diffuse SSc	CD19	mRSS decline; first SSc case	Mild
Auth (2025, Lancet Rheumatol)	Case series	Diffuse SSc	CD19	Skin and visceral improvement	Mostly low-grade
Wang W (2024, Ann Rheum Dis)	Phase 1, open-label	SLE	BCMA-CD19 compound	Remission within ~3 months	Acceptable
Wang X (2024, Cell)	Series, n=3	Myositis/SSc	Allogeneic CD19	Efficacy without acute rejection	Low-grade
CASTLE (2026, Nat Med)	Basket trial	Multiple autoimmune	CD19	Cross-indication responses	Consistent with prior reports

**Table 3:** Cross-disease comparison of response characteristics to CD19 CAR T-cell therapy.

Dimension	SLE	IIM	SSc
Principal B-cell-dependent driver	Immune complexes/autoantibodies	Autoantibodies + muscle MHC-I	Pro-fibrotic B-fibroblast crosstalk
Hallmark autoantibody	Anti-dsDNA	Myositis-specific (e.g. anti-Jo-1)	Anti-Scl-70/topoisomerase I
Serologic response	Rapid seroconversion	Rapid antibody loss	Variable
Tissue/organ response	Glomerular histology improves	Muscle MHC-I down; CK normalizes	Skin softening; visceral fibrosis slower
Typical speed	Fast	Moderate	Slow (structural remodelling)
Representative readout	SLEDAI → 0; DORIS	Manual muscle test; CK	mRSS; HRCT/cardiac MRI

CASTLE, a phase 1/2 basket trial of CD19 CAR-T conducted across multiple autoimmune diseases; CRS, cytokine release syndrome; DORIS, Definition Of Remission In SLE; ICANS, immune effector cell-associated neurotoxicity syndrome; mRSS, modified Rodnan skin score; SLEDAI, SLE Disease Activity Index.

## 4 Redefining the safety spectrum

### 4.1 Cytokine release syndrome

Cytokine release syndrome (CRS) is the most characteristic acute toxicity of CAR-T, and its severity is graded according to the consensus criteria of the American Society for Transplantation and Cellular Therapy (ASTCT) (139–141). In cancer therapy, the enormous target-cell burden can provoke severe CRS, accompanied by high fever, hypotension, and even multi-organ failure. In rheumatic disease, however, CRS almost uniformly manifests as grade 1 and self-limiting, mostly with only low-grade fever and without the need for vasoactive drug intervention (142–144). The molecular basis is that the pathogenic B-cell burden in autoimmune disease is far lower than in cancer, so the cytokine release provoked by CAR-T expansion is correspondingly mild; in addition, the differences between autoimmunity and cancer in inflammatory context and immune-activation thresholds also render CAR-T activation more restrained in the former (145–147). This markedly milder CRS profile is an important prerequisite for the safe application of CAR-T in non-malignant diseases.

### 4.2 The rarity of immune effector cell-associated neurotoxicity syndrome

Immune effector cell-associated neurotoxicity syndrome (ICANS) is a serious complication to be vigilant for in oncology CAR-T therapy, but it is extremely rare in the application to autoimmune disease (148, 149). In the reported autoimmune case series, severe ICANS events are almost absent, consistent with the lower peak cytokine levels (150). Even so, given the potential severity of neurotoxicity, standardized neurological monitoring and graded management according to ASTCT criteria are still required during treatment.

### 4.3 Long-term immune compromise

The principal long-term safety concern of CAR-T is not acute toxicity but sustained immune compromise. After deep B-cell depletion there is a period of B-cell aplasia, with B cells in most patients beginning to reconstitute several months after treatment (usually around 6 months) (151–153). During the aplastic period, some patients develop hypogammaglobulinemia, and the indication for immunoglobulin replacement therapy should be assessed according to serum IgG levels and infection status (154). Analysis of the infection spectrum indicates that attention should focus on viral reactivation (such as varicella-zoster virus and cytomegalovirus) and common bacterial infections, with prophylactic antiviral therapy and, when necessary, immunoglobulin support being central to management (155, 156). Because the pre-existing protective antibodies maintained by long-lived plasma cells are largely preserved, vaccine-induced immune memory is usually not significantly affected, which mitigates some of the infection risk (83).

### 4.4 Secondary autoimmunity and long-term malignancy risk

In the oncology CAR-T field, in 2024 the U.S. Food and Drug Administration required a boxed warning regarding secondary T-cell malignancies to be added to the labels of all approved CD19/BCMA CAR-T products, and recommended lifelong monitoring (157). This signal should be viewed objectively: it derives from approximately two dozen events reported among tens of thousands of cancer patients, with a very low absolute incidence, and these patients had often previously received multiple lines of chemotherapy and radiotherapy, so the causal relationship remains unclear (158–160). The analysis by Marks and Verdun in the New England Journal of Medicine noted that such events are relatively rare and that the overall benefit still far outweighs the risk (157, 161). In the autoimmune population, owing to the limited cumulative number of treated cases and the short follow-up, the true risk of long-term malignancy and secondary autoimmunity still cannot be accurately estimated—according to the statistical “rule of three, “ even if no serious events are observed among several hundred cases, the existence of a low-frequency risk cannot be excluded (92). This uncertainty calls for the establishment of long-term, systematic follow-up registries.

### 4.5 Considerations for special rheumatic populations

**Figure 3:**
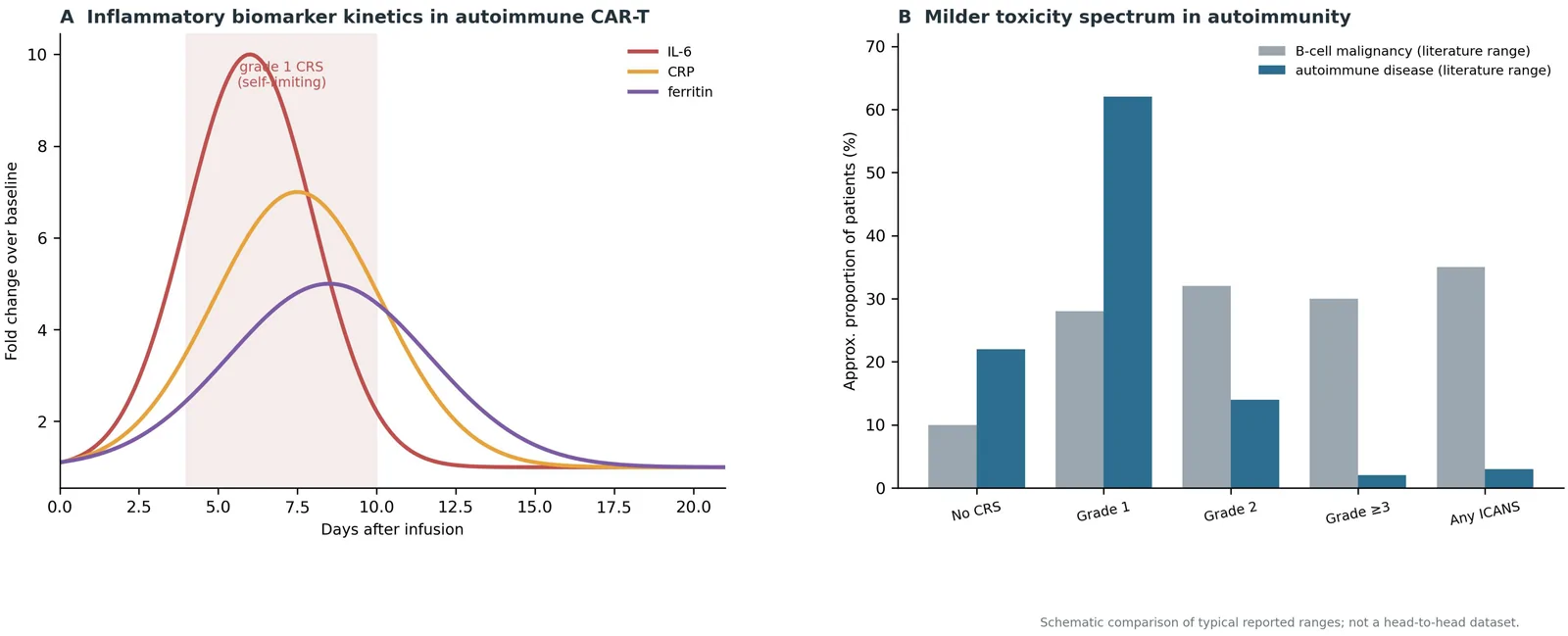
The redefined safety spectrum. **(A)** Inflammatory biomarker kinetics during a typical self-limiting grade 1 CRS. **(B)** Schematic comparison of toxicity-grade distribution between oncology and autoimmune settings.

Rheumatic patients often already have underlying damage to organs such as the heart, lungs, and kidneys, which differs from the situation of treatment-naive cancer patients and directly affects the safety window of CAR-T (117). For example, SSc patients with cardiopulmonary fibrosis tolerate the hemodynamic fluctuations associated with CRS less well; the baseline renal function of lupus nephritis patients influences dose adjustment of lymphodepleting agents; and a long history of immunosuppression alters the baseline infection risk (2). Therefore, the safety management of autoimmune CAR-T cannot simply copy the oncology experience but must perform individualized risk assessment and preconditioning optimization based on each patient’s organ reserve. The European Society for Blood and Marrow Transplantation (EBMT) and others have issued practice consensus statements on cellular therapy for autoimmune disease, providing a preliminary framework for the standardization of this emerging field (162, 163) (**Figure 3**). 

## 5 Next-generation engineering: toward precision immune modulation

### 5.1 Expanding the target dimension

**Figure 4:**
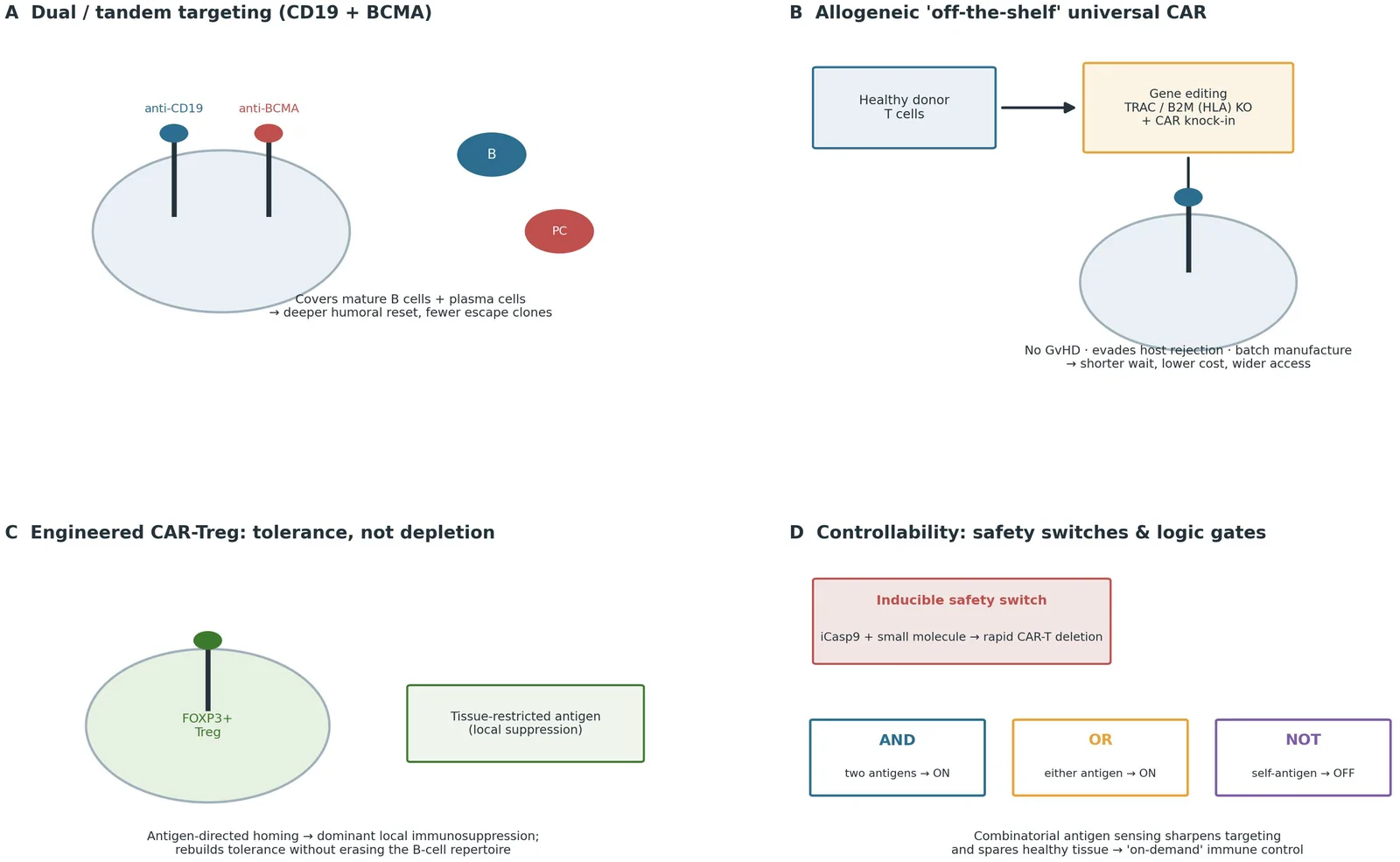
Next-generation engineering strategies for autoimmune CAR-cell therapy. **(A)** Dual/tandem targeting. **(B)** Allogeneic universal CAR. **(C)** Engineered CAR-Treg. **(D)** Safety switches and logic gates.

The first generation of autoimmune CAR-T was predominantly based on the single CD19 target, but CD19-negative long-lived plasma cells can serve as a persistent source of autoantibodies and a potential root of relapse (83, 164–167). Expanding the target dimension aims to achieve more thorough depletion: CD19/BCMA dual targeting (tandem CAR or co-infusion) can simultaneously cover the full spectrum of B cells and plasma cells (**Figure 4**), while CAR designs against plasma-cell-specific markers such as CS1 (SLAMF7) and CD38 further focus on the autoantibody “factory” (168, 169). Expanding the target requires a careful trade-off between the thoroughness of depletion and the preservation of humoral immunity. It should be emphasized, however, that direct comparative clinical evidence establishing the superiority of dual CD19/BCMA targeting over CD19-only CAR-T remains limited, as most available data derive from small single-arm cohorts rather than head-to-head studies. The additional depth of depletion also carries a plausible cost: broader elimination of the plasma-cell compartment may deepen and prolong hypogammaglobulinemia, increase infection risk, and further blunt vaccine responsiveness. Whether the incremental benefit of dual targeting outweighs this deeper and potentially more durable humoral immunosuppression is therefore an open question that will need to be resolved through controlled comparison.

### 5.2 Breakthrough in the source dimension: allogeneic universal CAR-T

Autologous CAR-T has a long manufacturing cycle and high cost and is constrained by the quality of the patient’s own T cells, which is especially prominent in chronically immunosuppressed rheumatic patients (170). Allogeneic universal CAR-T, by using gene editing to knock out the T-cell receptor (TCR, to avoid graft-versus-host disease) and human leukocyte antigens (HLA, to circumvent host rejection), achieves an “off-the-shelf” product from healthy-donor-derived cells, substantially shortening waiting time and lowering cost (171–178). Wang et al. reported in Cell the results of allogeneic CD19-targeted CAR-T in patients with severe myositis and systemic sclerosis, demonstrating that the gene-edited allogeneic product could circumvent acute immune rejection and exert efficacy, although residual alloreactivity may ultimately lead to clearance of the graft (179). Universal products are of strategic significance for moving CAR-T from specialized centers toward broader accessibility.

### 5.3 Reversing the functional dimension: engineered CAR-Treg

The aforementioned strategies all center on “depletion,” whereas CAR-Treg represents a fundamental reversal of the functional dimension—rebuilding homeostasis through “regulation.” Regulatory T cells (Treg) are key to maintaining self-tolerance, but polyclonal Treg therapy lacks antigen specificity (180). CAR-Treg, by endowing Tregs with the ability to recognize antigens specific to the diseased tissue, directs their migration to the lesion and exerts dominant local immunosuppression, achieving precise “cell-treats-cell” regulation (181–183). In the field of transplant tolerance, HLA-A2-targeted CAR-Treg has entered phase I/II clinical evaluation, and preclinical studies have shown that it can specifically localize to grafts expressing the target antigen and exert antigen-dependent suppressive function *in vivo* (184, 185). Gene editing at the FOXP3 locus to stabilize the Treg phenotype, together with the introduction of an inducible IL-2 signaling system, can further enhance the stability and function of engineered Tregs (186–188). The advantage of CAR-Treg is that it rebuilds tolerance without destroying the immune repertoire, theoretically avoiding the risk of long-term immunodeficiency; however, its phenotypic stability and whether it might destabilize and convert into effector cells in an inflammatory environment remain key problems to be solved.

### 5.4 Upgrading the control dimension: safety switches and logic gating

As engineering complexity increases, the demand for “controllability” of drug-laden cells *in vivo* grows accordingly. Safety switches such as the inducible caspase-9 (iCasp9) suicide gene can rapidly eliminate CAR-T by administering a small-molecule inducer when severe toxicity occurs, while gating systems based on antibody epitopes or tyrosine kinases provide a means of pharmacologically regulating CAR-T activity (189–191). Logic-gated CARs—including AND gates (activation requires simultaneous recognition of two antigens), OR gates (recognition of either antigen activates), and NOT gates (recognition of a normal-tissue antigen suppresses activation)—improve targeting precision through combinatorial antigen recognition and reduce off-target toxicity (192–194). In the autoimmune setting, such precise control systems hold promise for achieving “on-demand” immune modulation: clearing pathogenic cells while maximally preserving normal immune function. In addition, *in vivo* CAR-T technology—using targeted lipid nanoparticles (LNP) to deliver CAR-encoding mRNA and generate transiently expressed CAR-T directly within the patient—is emerging, and its freedom from ex vivo manufacturing and lymphodepletion may fundamentally change the accessibility and safety of CAR-T (195, 196). In parallel, extracellular vesicle-based mRNA delivery is being explored as a complementary *in vivo* route for encoding CAR machinery, potentially broadening the delivery toolbox beyond lipid nanoparticles (197). Preclinical studies have already demonstrated that this strategy can generate CAR-T *in vivo* and reverse fibrosis or reset B cells (198–200).

## 6 Challenges and future horizons

### 6.1 Which patients are best suited for CAR-T?

**Figure 5:**
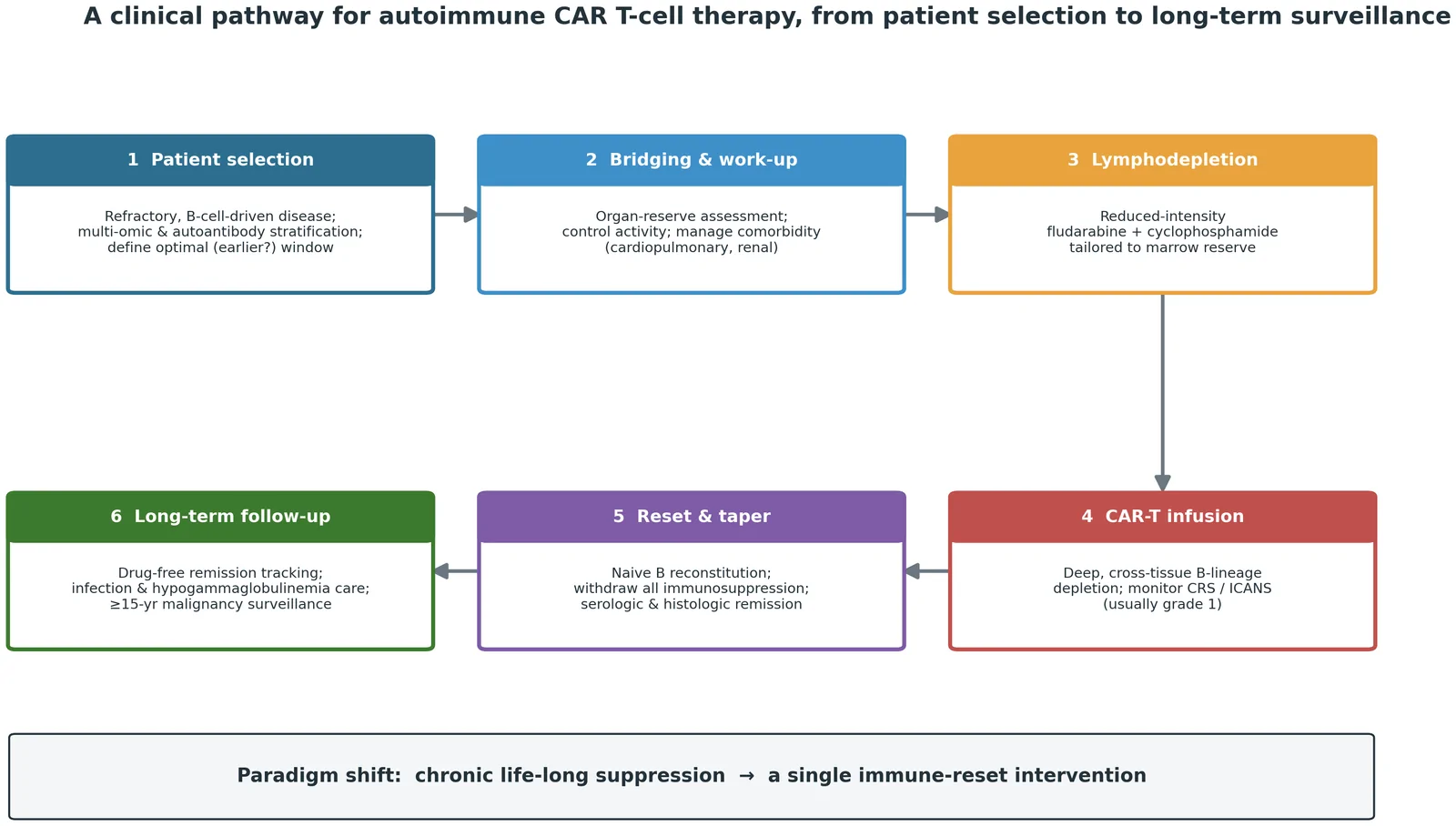
A clinical pathway for autoimmune CAR T-cell therapy, from patient selection to long-term surveillance.

CAR-T is a potent intervention but one of considerable intensity and cost, making rational patient selection essential (**Figure 5**). Current application is mostly limited to refractory, life-threatening patients who have failed multiple lines of therapy, consistent with a “last-line salvage” positioning (162). However, intervening early—before irreversible damage has formed—may yield better organ protection and reset effects. Precision stratification based on multi-omics (genome, transcriptome, B-cell receptor repertoire sequencing, autoantibody profiling) holds promise for identifying the population most likely to benefit from deep B-cell depletion with manageable relapse risk, and for defining the optimal early intervention window (201). How to strike a balance between “overtreatment” and “missing the window” is a core dilemma of clinical decision-making.

### 6.2 Does “drug-free remission” equal “cure”?

The drug-free remission brought about by CAR-T is exciting, but whether it is equivalent to a “cure” still requires rigorous definition. The durability of immune reset is key: as the B-cell pool reconstitutes and immune function recovers, will autoimmunity relapse after a sufficiently long time? If relapse is extremely rare, it would suggest that these diseases may originate from specific triggers that misdirect the immune system, rather than purely from genetic and hormonal susceptibility—which would have profound etiological significance (46). Conversely, if some patients relapse, it would be necessary to identify the microenvironmental factors that may reignite autoimmunity (such as residual autoreactive T cells or persistent antigen exposure within tissues). Therefore, any claim of “cure” must be built on long-term follow-up data, and it is premature to draw such a conclusion at present. This caution is reinforced by the still-limited duration of follow-up in most reported series, which rarely extends beyond two to three years. It thus remains uncertain whether the drug-free remission observed so far reflects durable disease control or whether late relapse may yet emerge, for instance through immune reconstitution, epitope spreading, or the re-emergence of autoreactive clones from residual precursors. Substantially longer longitudinal data will be required before any claim of durable control, let alone cure, can be justified.

### 6.3 Health economics and healthcare equity

The high one-time cost of CAR-T is a practical obstacle to its broader adoption. However, from a whole-course-cost perspective, it must be compared in overall cost-effectiveness against the lifetime cumulative costs of chronic treatment, repeated hospitalizations, replacement therapy after organ damage (such as dialysis), and lost productivity in rheumatic patients (202). If CAR-T can replace decades of chronic management with a single treatment, its long-term economic value may be better than intuitive judgment suggests. If allogeneic universal and *in vivo* CAR-T technologies can mature, they would significantly lower the unit cost and are key to improving healthcare equity and bringing this therapy to a broader population (203). Innovations in health policy and payment models (such as outcomes-based payment) also need to develop in tandem.

It is also instructive to benchmark CAR-T not only against chronic drug therapy but against emerging cell-directed alternatives, in particular BCMA-directed T-cell engagers, which have already shown activity in refractory autoimmune disease (138). T-cell engagers are off-the-shelf biologics that dispense with ex vivo manufacturing, lymphodepleting preconditioning, and the associated inpatient stay, and are therefore likely to be cheaper and easier to deliver at the point of care; natural killer (NK)-cell platforms share several of these logistical advantages. The corresponding limitation is the mirror image of this convenience: because they do not durably remodel the B-cell repertoire, they generally require indefinite repeat dosing to sustain their effect. The economic comparison is thus one of a large one-time expenditure that may purchase a durable immune reset versus a lower but recurring lifetime cost that does not. This distinction bears directly on the paradigm shift argued for in this review and should be weighed alongside the oncology-derived cost-effectiveness estimates on which the preceding discussion has necessarily relied.

### 6.4 The trial pathway from “last-line salvage” to “first-line cure”

Moving CAR-T from the last line toward earlier lines and even the first line requires a paradigm shift in clinical trial design. This calls for randomized controlled trials with endpoints such as the duration of drug-free remission, progression of organ damage, and quality of life, conducted in head-to-head comparison with existing standard therapy and incorporating health-economic evaluation (204, 205). At the regulatory level, regulators in the United States and Europe are exploring pathways for autoimmune CAR-T that balance flexibility with long-term safety monitoring, for example requiring follow-up of more than a decade to monitor long-term events (157). The construction of multicenter, standardized patient registries and biobanks will provide support for building this body of evidence.

## 7 Conclusion

CAR-T therapy has rapidly moved from a single proof-of-concept case to center stage in the treatment of rheumatic and autoimmune disease. Through deep, cross-tissue depletion of pathogenic B-lineage cells and the subsequent naive-repertoire-dominated immune reconstitution, CD19 and BCMA CAR-T have achieved—in systemic lupus erythematosus, idiopathic inflammatory myopathy, and systemic sclerosis—sustained remission, autoantibody seroconversion, and reversal of tissue pathology after withdrawal of all immunosuppressants, a “depth” unattained by any previous B-cell-targeted therapy. In the autoimmune setting, with its lower target burden, CAR-T exhibits a safety profile markedly milder than in cancer therapy, yet long-term immune compromise, infection management, and long-term malignancy risk still need to be defined through disciplined long-term follow-up. Next-generation engineering advances—represented by dual targeting, allogeneic universal products, CAR-Treg, safety switches and logic gating, and *in vivo* CAR-T—are simultaneously broadening indications, lowering costs, and enhancing precision and controllability. While this therapy redefines the boundaries of “remission” and “cure,” the field still needs to answer fundamental questions about durability, precise patient stratification, health economics, and trial pathways. It can be foreseen that, as randomized controlled evidence accumulates and engineering technologies mature, CAR-T is poised to drive a historic shift in the rheumatic-disease treatment paradigm from “chronic suppression” toward a “one-time immune reset.”
